# Immune Subtyping in Latent Tuberculosis

**DOI:** 10.3389/fimmu.2021.595746

**Published:** 2021-04-07

**Authors:** Ushashi Banerjee, Priyanka Baloni, Amit Singh, Nagasuma Chandra

**Affiliations:** ^1^ Department of Biochemistry, Indian Institute of Science, Bangalore, India; ^2^ Centre for Infectious Disease Research, Indian Institute of Science, Bangalore, India; ^3^ Center for Biosystems Science and Engineering, Indian Institute of Science, Bangalore, India

**Keywords:** latent tuberculosis, genome-wide network analysis, transcriptomics, heterogeneity, immune subtypes

## Abstract

Latent tuberculosis infection (LTBI) poses a major roadblock in the global effort to eradicate tuberculosis (TB). A deep understanding of the host responses involved in establishment and maintenance of TB latency is required to propel the development of sensitive methods to detect and treat LTBI. Given that LTBI individuals are typically asymptomatic, it is challenging to differentiate latently infected from uninfected individuals. A major contributor to this problem is that no clear pattern of host response is linked with LTBI, as molecular correlates of latent infection have been hard to identify. In this study, we have analyzed the global perturbations in host response in LTBI individuals as compared to uninfected individuals and particularly the heterogeneity in such response, across LTBI cohorts. For this, we constructed individualized genome-wide host response networks informed by blood transcriptomes for 136 LTBI cases and have used a sensitive network mining algorithm to identify top-ranked host response subnetworks in each case. Our analysis indicates that despite the high heterogeneity in the gene expression profiles among LTBI samples, clear patterns of perturbation are found in the immune response pathways, leading to grouping LTBI samples into 4 different immune-subtypes. Our results suggest that different subnetworks of molecular perturbations are associated with latent tuberculosis.

## Introduction

*Mycobacterium tuberculosis* (*Mtb*) is one of the most successful pathogens known to humans. Despite the availability of an array of anti-mycobacterial drugs, tuberculosis (TB) is still the leading cause of death among infectious agents. Although it is the active form of TB that causes morbidity, contagiousness and mortality, a majority of *Mtb* infected individuals remain latently infected (1). These individuals, accounting for approximately 1.7 billion people worldwide, harbor the dormant pathogen while remaining clinically asymptomatic for decades and carry a 10% lifetime risk of TB reactivation and thus act as reservoirs of the TB bacilli (1, 2). Therefore to eradicate TB, it is not only necessary to treat the active TB patients, but also important to successfully diagnose and treat LTB infection (LTBI) in asymptomatic individuals. Establishment and maintenance of latency result from an equilibrium in the host-pathogen interactions where the host immune response can successfully contain the spread of the bacteria by forming granulomatous lesions but fails to completely eradicate it (3).

Multiple studies have previously shown the presence of a wide spectrum of disease in tuberculosis, including latent, incipient, subclinical and active TB (4, 5). Although the last three types have historically gathered more research focus due to the imminent threat to the patient, it is important to gain deeper understanding into the mechanisms that establish and maintain LTBI condition, preventing its progression to further stages.

Multiple challenges are involved in studying LTBI. At the outset, accurate diagnosis of LTBI remains to be problematic as the Interferon Gamma Release Assay (IGRA), the most sensitive technique currently available, lacks the specificity to differentiate between active and latent TB as well as new and treated infections (6). Moreover, as LTBI individuals are clinically asymptomatic and do not routinely require any clinical intervention, testing is often limited to primary contacts of active TB patients. Next, there is a lack of an ideal animal or *in vitro* model for LTBI, making it hard to study the condition. Studies involving whole blood samples from LTBI subjects can be expected to throw more light on the precise host immune response in maintaining latency of *Mtb*. Systems level studies based on host transcriptome data are increasingly being used to gain a holistic insight into different perturbations in TB and other infectious diseases (7–9). These studies have been successful in gaining mechanistic insights into active TB (10–12) as well as in identifying biomarkers to differentiate active TB from LTBI or uninfected individuals (7, 13–17). Blood-based transcriptomic signatures have also been successful in identifying the prospect of reactivation of LTBI (18). Recently, Burel and co-workers used transcriptomics and protein profiling of CD4+ T cells and identified an LTBI specific signature to separate them from uninfected cases (19). However, accurately differentiating non-incipient and non-subclinical LTBI from uninfected individuals has remained an open question (20).

In this work, we seek to obtain an unbiased and personalized view of the key immune responses that are either activated or repressed in response to a latent TB infection as compared to uninfected individuals. Towards this, we built sample-specific network models of the immune processes by using a computational pipeline previously developed in the laboratory that integrates transcriptomes of LTBI individuals with a human protein-protein interaction network to make precision networks for each subject. Whole blood transcriptomes for LTBI subjects were publicly available generated in multiple different studies. Our network that was recently reconstructed in the laboratory (21), is knowledge-based and covers interactions of proteins coded by the whole genome, where the interactions and functional influences are encoded with direction information. Our analysis algorithm sensitively mines these transcriptome-integrated networks to find the most important perturbations in LTBI and computes top-ranked perturbed subnetworks. We observed that although the gene expression profiles of the LTBI individuals vary greatly from the uninfected samples, there is a high amount of heterogeneity among the LTBI samples. Our analysis revealed that the highly heterogeneous gene expression profiles are related to perturbations in a limited number of pathways, belonging mostly to innate and adaptive immune responses. It further identified that the meta-cohort studied here could be stratified into immune subtypes where each subtype showed a unique perturbation pattern that arises from different combinations of these pathways, and also the subtype which might help in better clinical characterization. Our observations suggest that different molecular perturbations could be associated with the maintenance of TB latency by the host system.

## Methods

### Selection of Publicly Available Datasets

We searched the Gene expression repository GEO (22) for microarray and high throughput sequencing based expression profiling datasets on latently infected human samples using the keyword search ‘(Tuberculosis) AND. “Homo sapiens”[porgn:_txid9606]’. Using this search criteria we obtained a list of 196 datasets (both microarray and RNA-seq data) that we filtered using the following criteria: i) presence of LTBI as well as uninfected control samples, ii) data obtained only from whole blood and not cell type-specific, iii) absence of any comorbidity such as HIV, iv) more than 3 samples for each condition and v) cohort from an adult age group (over 18 years). 5 datasets matched the selection criteria. The microarray datasets, which also included samples from active TB patients were used as discovery datasets in this study. Active TB patients were identified with sputum smear, chest X-ray and/or culture positivity tests, whereas the LTBI cases were Tuberculin Skin Test (TST) or IGRA positive and sputum smear or culture test negative. Details of the 5 transcriptomic datasets utilized in this work are provided in **Table 1**. Further, 4 additional transcriptomic datasets (**Table 1**) were selected which included samples from LTBI individuals but not uninfected cases. These datasets were also used as a second layer of validation.

**Table 1:** Blood transcriptome datasets obtained from GEO satisfying the inclusion criteria as mentioned in *Methods* section.

GEO ID	Platform	Uninfected Samples	LTBI Samples	Active TB Samples	Geographic Location	Age Group	Dataset Usage	Reference
GSE19439	Illumina GPL6947	12	17	13	London, UK	Adult	Discovery	(7)
GSE19444	Illumina GPL6947	12	21	21	London, UK	Adult	Discovery	(7)
GSE28623	Agilent GPL4133	37	25	46	The Gambia	Adult	Discovery	(13)
GSE107993	Illumina HiSeq GPL20301	15	16	–	Leicester, UK	Adult	Validation	(23)
GSE107994	Illumina HiSeq GPL20301	50	57	–	Leicester, UK	Adult	Validation	(23)
GSE37250	Illumina GPL10558	–	83	97	South Africa, Malawi	Adult	Validation (Set 2)	(14)
GSE40553	Illumina GPL10558	–	36	29	South Africa	Adult	Validation (Set 2)	(24)
GSE79362	Illumina Hiseq GPL11154	–	153	–	South Africa, Gambia	Adolescent	Validation (Set 2)	(18)
GSE101705	Illumina Nextseq GPL18573	–	16	27	South India	Adult	Validation (Set 2)	(25)

### Processing of Gene Expression Data and Statistical Analysis

We obtained raw data for each dataset from the GEO. The datasets were pre-processed and normalized separately since the platform chemistry was different for each dataset. EdgeR and Limma package in Bioconductor in the R statistical environment was used for all gene expression analysis (26–28). Overall, each dataset was subjected to background correction, normalization and probe-set summarization. The normalized and log2 transformed gene expression values were used for calculation of differential expression between conditions, with moderated t-statistics and Benjamini-Hochberg’s method to control the false discovery rate.

In case of the datasets with only LTBI samples but not uninfected cases, the gene expression profiles were computed in comparison to the uninfected samples from other datasets. The tool ComBat (29) was used to merge the normalized gene expression data from individual datasets and remove batch effect, followed by differential gene expression calculation with the Limma package.

### Generating Precision Networks for Each LTBI Subject

We used a comprehensive in-house human protein-protein interaction network (hPPiN) from Sambarey et al., 2017 in this study (21). In summary, the network contained high confidence, experimentally validated physical and functional interactions curated from different databases, mainly STRING (30), SignaLink 2.0 (31), BioGRID (32) and primary literature (10, 33). The proteins in the hPPiN were represented as ‘nodes’ and the interactions between the proteins were represented as ‘edges’. The edges were given directions based on the nature of interaction between the proteins. While the edges signifying interactions like activation, inhibition, phosphorylation, etc. were marked as unidirectional, the physical binding interactions and interactions without functional annotation were marked as bidirectional. The network comprises 17,070 nodes and 209,582 edges, of which 80% of the edges are directed, which is used as the base network. We converted the base network to individualized precision networks for each LTBI subject by integrating it with the corresponding subject’s transcriptome data. For this, we used an in-house computational approach (10, 17, 21, 34) that derives node and edge weights as per Equations 1, 2 and 3. In every case LTBI samples were compared against the median gene expression values of uninfected samples from the same dataset.

To mine the weighted LTBI networks, we use our previously developed computational method (10, 17, 21, 35), which involved identification of ‘most active’ and ‘most repressed’ paths in each network based on the path cost. Path cost was computed as the sum of weights of edges present in the path normalized by the number of nodes in the path (Equation 4). The least cost paths in each sample encompassing the top 500 nodes were selected as the most active and repressed paths. Networks obtained from most-active paths formed the top-active network (LTBINetA) and the most-repressed paths constituted the top-repressed network (LTBINetR). LTBIA and LTBIR were combined to generate the top-perturbed precision network (LTBINetP) for each individual.

### Functional Enrichment Analysis

Pathway enrichment analysis of the precision networks, LTBINetP, was carried out with Enrichr (36). Enrichment of immune response pathways was carried out in higher detail by using InnateDB, the manually curated innate immunity pathway and interactions database (37) with a hypergeometric test and the Benjamini-Hochberg’s correction method. For both Enrichr and InnateDB enrichment, pathways with corrected P-value ≤0.05 were considered to be significantly enriched.

Since the pathway description in the databases includes all the genes involved in the pathways and many pathways share multiple signal mediators, there is a significant overlap of genes between different pathways. Therefore, a pathway can be statistically over-represented if some of the signal mediators are captured in the gene list, but not the initiating genes of the pathway, i.e. the ligands or receptors. In order to avoid the selection of such pathways from further analysis, another curation step was applied to the enrichment analysis to select only those pathways for which the ligand or receptor genes (as per the source databases) were present in the LTBINet analyzed.

### Computation of Binary Pathway Over-Representation Score and Clustering

Each of the target pathways was given a binary score of 1 or 0 based on the over- representation of the pathway associated genes (corrected p-value ≤1e-05) in the top perturbed network of individual samples. The precise pattern of pathway perturbation was used for clustering the samples. Unsupervised clustering of LTBI samples was performed with the pathway perturbation scores using the ‘ConsensusClusterPlus’ package in R (38).

## Results

### A Meta-Analysis of Whole Blood Transcriptomes of LTBI Individuals Reveal Heterogeneity in Gene Expression

**Figure 1:**
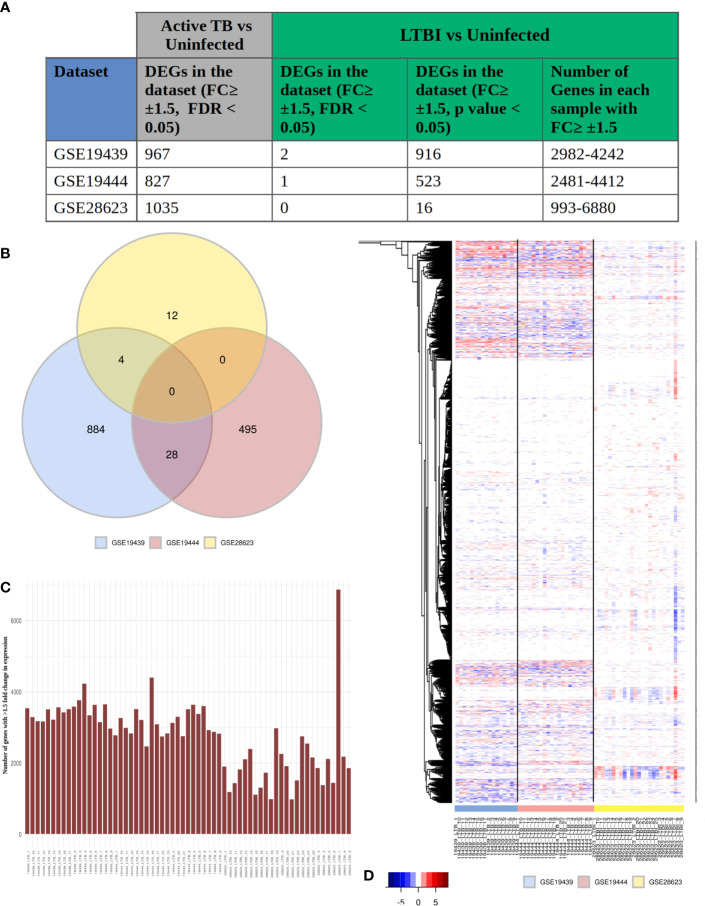
LTBI patients have a highly heterogeneous gene expression profile. **(A)** The three selected datasets GSE19439, GSE19444 and GSE28623 showed a significant number of genes to be differentially regulated in active TB condition but very few DEGs in LTBI when compared to uninfected cases with conventional thresholds of FDR ≤0.05 and fold change ≥ ± 1.5 criteria. More number of genes could be considered as DEGs if the criteria are modified to unadjusted p value ≤0.05 and fold change ≥ ± 1.5. However each of these samples contained thousands of genes with ≥ ± 1.5 fold change in gene expression compared to uninfected individuals. **(B)** Venn diagram shows that there are no common DEGs in LTBI condition (unadjusted p value ≤0.05, fold change ≥ ± 1.5) between all three datasets. The number of common DEGs between any two datasets are also very few. **(C)** Each LTBI sample contained over 1000 genes with ≥ ± 1.5 fold change, showing a significant difference between the gene expression profile of the individual samples from uninfected cases. **(D)** Heatmap shows the fold change of expression of the genes in each individual LTBI sample that showed (≥ ± 1.5) fold change in any sample (union of all genes from **Figure 1A**, column 5). White signifies no significant fold change (≤ ± 1.5), red stands of upregulation in gene expression and blue shows downregulation. It is clear that although all the samples show significant change in the gene expression profiles, it is not consistent across the samples, suggesting a heterogeneous response to LTBI in humans.

To obtain a systems perspective of the host immune responses associated with LTBI, we first analyzed 3 publicly available transcriptome datasets, GSE19439, GSE19444 and GSE28623, that contained information on samples from LTBI, uninfected healthy controls (HC) as well as active TB cases and subsequently validated our findings in 2 other independent LTBI transcriptome datasets (GSE107993 and GSE107994). The 3 datasets together contained samples from 63 LTBI (TST or IGRA +ve, sputum smear or culture test -ve) and 55 healthy individuals. We found very few differentially expressed genes (DEGs) between LTBI and HC, with a fold change ≥ ± 1.5, FDR adjusted p value ≤0.05 (2 in GSE19439, 1 in GSE19444 and 0 in GSE28623) (**Figure 1A**), although a significant number of genes (1000) were found to be differentially regulated (fold change ≥1.5, FDR adjusted p value ≤0.05) between active TB and HC in all 3 datasets (**Figure 1A**). However, with less stringent statistical criteria (unadjusted p value ≤0.05 for the same fold change cut-off of ≥ ± 1.5), there was a significant increase in the number of DEGs in each dataset [ranging from 993 to 6680 (**Figure 1C**)], but there were no common DEGs among them (**Figure 1B**). Analysis of the DEG lists indicated that transcriptomes in LTBI samples do exhibit many variations as compared to HC (**Figure 1A**), but the DEG lists differ greatly between cohorts and also within each cohort, but the same genes were not differentially expressed across all LTBI samples and the extent of differential expression of the genes was also not similar (**Figure 1D**). This clearly shows that there is a high amount of heterogeneity amongst the LTBI samples, which leads to the absence of statistically significant DEGs when many samples are analyzed together in or across datasets.

### Genome-Wide Response Network Analysis Identifies Most Frequently Perturbed Pathways in LTBI Individuals

**Figure 2:**
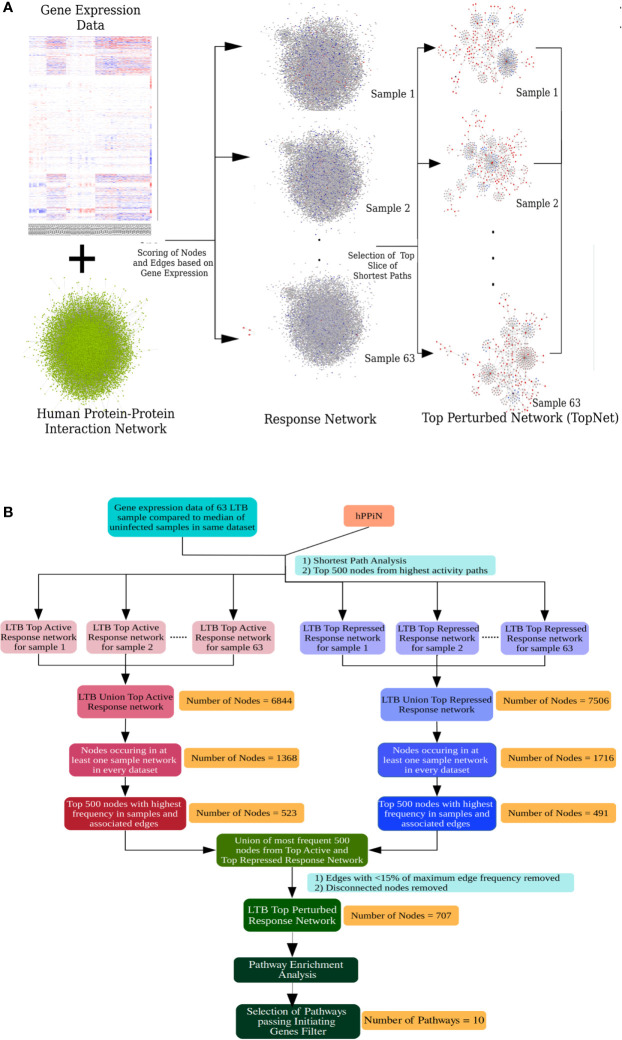
Response network analysis workflow. **(A)** Schematic representation of the individualized protein-protein interaction network analysis workflow used in this work. **(B)** Workflow to identify the most frequently perturbed immune response pathways in LTBI patients.

The inherent heterogeneity in the gene expression profile of LTBI individuals makes a conventional differential gene expression analysis miss out on several genes that may play an important role in the host response in some LTBI individuals but not all. At a functional level, a pathway might be perturbed as a result of differential regulation in any of the key genes involved in the pathway, leading to a similar end-effect at the level of phenotype. To understand if the highly heterogeneous gene expression profile of the LTBI individuals were indeed involved in perturbing a similar group of pathways, it became necessary to study the effect of the gene expression in each case at a functional level. A network approach involving genome-wide protein-protein interaction networks integrated with condition specific gene expression data has been shown to be more efficient in studying underlying cross-talks between DEGs and identifying functional alterations between the two conditions (21, 35, 39). We have previously developed methods in our laboratory to construct condition-specific networks by incorporating transcriptome data with a genome-wide protein-protein interaction network and further to sensitively mine such networks to identify subnetworks containing top-ranked perturbations in the condition of interest (21, 34). We adopted this approach to build LTBI specific response networks for each of the 63 samples. Briefly the method integrates the in-house human protein-protein interaction network (hPPiN) with gene expression data and identifies a connected set of perturbed paths (as compared to HC), from which the top 500 nodes in each case is taken to constitute a top-ranked response network (LTBINets) (**Figure 2A**). Each of the 63 LTBINets contained one large connected component containing genes belonging to broadly similar functional categories in each case. An enrichment analysis based on the Reactome Database showed that the predominant pathways were those of the immune response, signal transduction and cell cycle phases (**Supplementary File S2.A**). This clearly indicated that, despite a lack of common DEGs across these samples, the LTBINets contained genes of the same functional categories, showing that they shared commonalities in the alteration patterns at the level of pathways.

We focused on studying the most important immune response pathways and used InnateDB database for further over-representation analysis. However, we perceived that the ontologies that describe the pathway associations for genes are rather broad and multiple pathways share a large number of overlapping genes. Thus, a pathway can be statistically enriched despite the lack of the key genes in the input that trigger the pathway, leading to a possibility of false positives. To address this problem, we applied a filter that checks for feasibility in pathway perturbation by eliminating all those where the initiating genes of the pathway were absent. Specifically, we obtained a frequency-weighted union of all 63 LTBINets and pruned the pooled network to retain only those nodes that were present in at least 20% of LTBINets (**Figure 2B**) and studied the most enriched immune response related pathways that satisfied our criteria of the presence of initiating genes (**Supplementary File S2.C**). We further manually curated the list of over-represented pathways to identify the specific immune responses important in LTBI. From the list of pathways in **Supplementary File S2.C** only those pathways were retained that a) represented a specific cellular signaling or immune response pathway, and b) satisfied our criteria of the presence of pathway initiating genes (i.e. ligands or receptors) as described in the corresponding databases in the union LTBINet (**Supplementary File S2.D**). Thereby 13 pathways, comprising 10 distinct immune response signaling pathways were obtained from the pruned network that could be clearly linked to LTBI in most cases and refer to these as Immune response pathways in LTBI (IPLTB). These are the IFNγ mediated signaling, signaling by IL2, IL4 and IL12, TNFα and TGFβ signaling pathways, TLR2 mediated signaling and the signaling pathways by receptor tyrosine kinases EGFR, PDGF and FGFR. All of these pathways except TNF, IL2 and IL4 mediated signaling were found to be more active (more frequent in top active networks) in LTBI cases, whereas IL4 signaling activity was found to be repressed (more frequent in top repressed networks) in LTBI as compared to HC (**Supplementary File S2.E**). TNFα and IL2 pathways showed higher activity in majority but lower in some LTBI individuals than HC. Most of these pathways have been reported to play significant roles in active TB, whereas interferon-γ, IL2 and TNFα mediated signaling have been clearly implicated in LTBI and host susceptibility to TB (**Supplementary Table S1.A**). Further, there are reports of reactivation of LTBI in cancer patients treated with receptor tyrosine kinase inhibitors, providing additional support for the involvement of these pathways in maintenance of latency (40, 41).

### Clustering of Samples Based on Perturbed Pathways Indicates Immune Response Sub-types in LTBI Individuals

**Figure 3:**
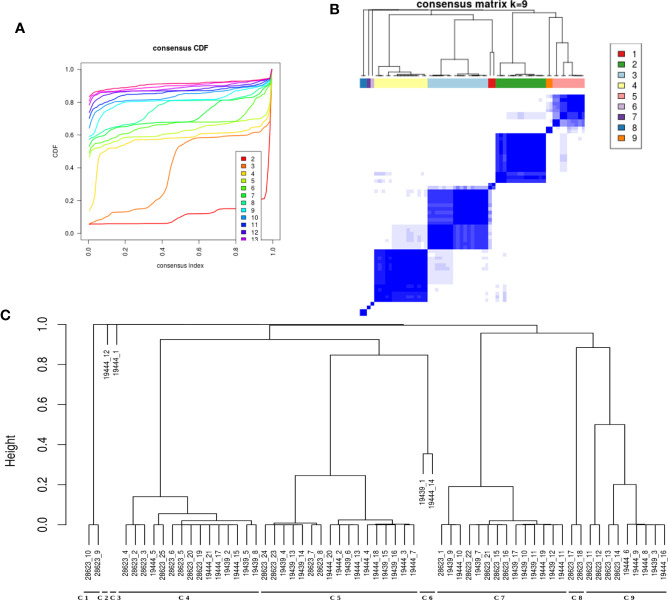
LTBI samples can be divided into groups based on pathway perturbation. **(A, B)** The LTBI samples can be divided into 9 substantially stable clusters based on their pathway perturbation patterns. The cumulative distribution function (CDF) plot shows that CDF reaches an approximate maximum as early as k=9 cluster. The clusters are significantly different from each other. 4 of the clusters contain the majority of the samples, whereas the few other samples show a highly varied pathway profile. **(C)** The dendrogram shows the samples in each cluster. The clustering was not biased by cohort or dataset as each of the large clusters contains samples from different datasets.

**Figure 4:**
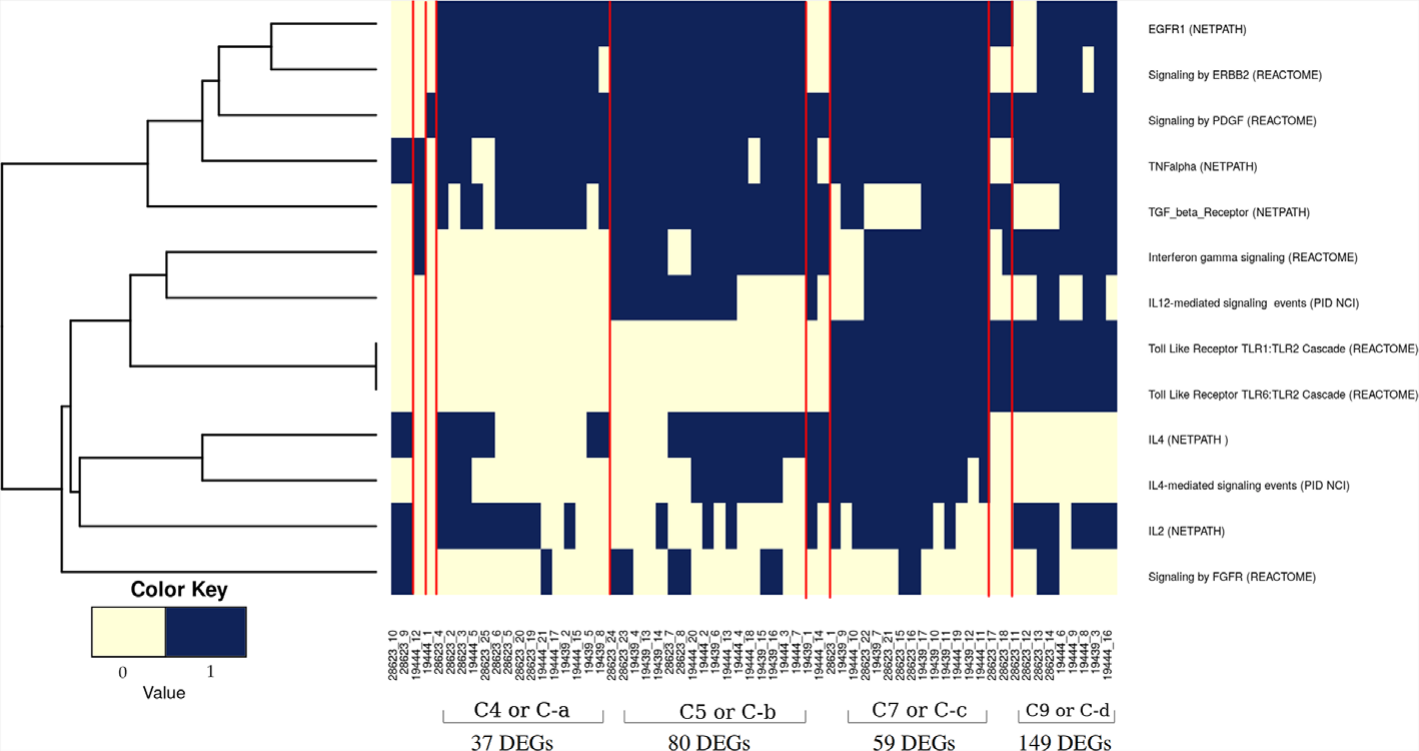
Pathway perturbation pattern in sample clusters. The pattern of the perturbations in each sample arranged according to their clusters (as mentioned in **Figure 3C**) is depicted in a binary scoring manner. Blue signifies that the pathway is perturbed in the patient and light yellow signifies no perturbation. Red lines demarcate the clusters.

It was evident from the network analysis that not every LTBI individual had perturbation in all the over-represented pathways. We therefore asked if there were any distinct subtypes among LTBI individuals in terms of their immune responses. To address this, we considered the most frequently perturbed immune pathways (IPLTB) and represented each sample as a binary barcode of perturbations of IPLTB (**Supplementary File S2.F**). We then clustered the samples based on the extent of similarity in the barcodes with an unsupervised clustering tool ConsensusClusterPlus with Euclidean distance metrics. The cumulative distribution function reached an approximate maximum at 9 clusters (C1 to C9), of which 4 clusters (C4, C5, C7 and C9) were large consisting of 87% of the total samples, whereas the rest of the samples formed small clusters clearly different from the rest (**Figures 3A, B**). The clusters were not dataset-specific, as samples from different datasets were found in the same cluster (**Figure 3C**). From here onwards, the major clusters C4, C5, C7 and C9 will be called C-a, C-b, C-c and C-d respectively. Almost all samples showed perturbation in the EGFR, PDGFR, TNFα and TGFβ signaling pathways, whereas the other pathways were perturbed only in a fraction of the LTBI samples. All samples belonging to the cluster C-a, show perturbations in PDGF, EGF, TGFβ and TNFα mediated signaling pathways, along with perturbed IL2 and IL4 mediated signaling in some cases. Samples in C-b show perturbations in IL12/IFNγ axis and IL4 mediated signaling in addition to the pattern in C-a. Samples in cluster C-c have the maximum perturbations since all but FGFR mediated signaling pathways are found to be perturbed in most of them. C-d significantly differs from C-c in not having any perturbation in IL4 mediated signaling and from C-a and C-b by showing variation in TLR2 mediated signaling. The other 9 samples have fewer perturbed pathways than the other clusters (**Figure 4**). We earlier observed the lack of common DEGs across all the samples from the datasets. Now we performed a gene expression analysis for samples in each of the four major clusters and saw that the number of DEGs (fold change ≥1.5, p value ≤0.05) was increased significantly in each cluster. C-a showed 37 DEGs, whereas C-b, C-c and C-d showed 80, 59 and 149 DEGs respectively (**Figure 4**). This shows that the sample heterogeneity is lesser within each subtype and the extent of perturbation also differed between the subtypes.

**Figure 5:**
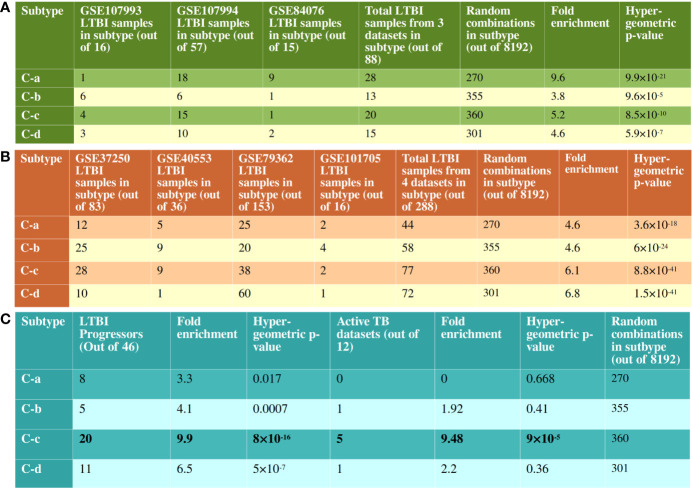
Validation of clustering pattern with independent RNAseq datasets. **(A)** There are 8192 possible combinations of binary scoring patterns for the 13 pathways used for clustering analysis. 14 out of 16 samples from GSE107993, 49 out of 57 samples from GSE107994 and 13 out of 15 samples from GSE84076 clustered into one of C-a, C-b, C-c or C-d. This is significantly more enriched than the random possibilities, validating the clustering pattern of pathways. **(B)** Similarly, in datasets without uninfected samples, 251 out of 288 samples clustered with one of the recognized immune subtypes. Notably, in GSE79362, 153 non-progressors were analyzed and 60 of them clustered with C-d, showing a bias for non-progressor for this subtype. **(C)** 44 of the 46 LTBI progressor samples from GSE79362 and GSE107994 clustered one of the 4 subtypes, with a clear bias towards C-c. 5 of the 12 active TB datasets also showed immune perturbation patterns similar to the subtype C-c.

We performed a 2 fold validation of the clustering patterns. In the first step, we analyzed the 2 RNAseq based transcriptome datasets that contained samples from both LTBI and uninfected cases. There were a total of 73 LTBI samples in GSE107993 and GSE107994 to which a similar network analysis pipeline followed by an enrichment analysis was applied. For each sample, a binary score was computed for each of the 10 immune pathways. Each of the 73 samples thus scored were added to the binary score table of the 63 previous samples and consensus clustering was performed. 63 of the 73 LTBI samples clustered with one of the four major clusters (C-a, C-b, C-c and C-d) showing the patterns to be significant for LTBI condition (**Figure 5A**). Similar to the discovery datasets, C-a contained a minimum number of DEGs (fold change ≥1.5, p value ≤0.05), which is 54, whereas C-d showed the highest number of DEGs, 325. C-b and C-c had 229 and 134 DEGs respectively (**Supplementary Figure 1**). The significant difference in the validation set samples from the previous set was that a higher frequency of perturbations in FGFR mediated signaling pathway was observed in all clusters and the higher frequency of perturbation in IL-2 mediated signaling in C-b among validation samples (**Supplementary Figure 1**).

In the second validation step, clustering patterns of the LTBI samples from the datasets lacking uninfected samples were analyzed in a similar manner. The gene expressions were calculated with respect to uninfected samples from other datasets as mentioned in the *Methods* section. These four datasets contained a total of 288 LTBI samples, of which 251 clustered with one of the four major clusters with significant hypergeometric p-value (**Figure 5B** and **Supplementary Figure 2**). These validation analyses clearly suggest that the host immune system in majority of the LTBI individuals follow one of the 4 identified subtypes.

As evident from **Figure 4** and **Supplementary Figures 1** and **2**, no pathway was perturbed in every sample, nor did any sample have perturbation in all pathways. Thus each of the clusters showed specific immune response subtypes associated with TB latency, of which 4 subtypes (C-a, C-b, C-c and C-d) are more frequently observed. This indicates that different individuals with the same phenotype of latent TB are associated with different genotypes for either establishing or maintaining latency. The samples in each cluster have similar perturbation (and similar genotypes) among them and hence form a subtype. The analysis clearly shows multiple subtypes, indicating that latency could be maintained by different molecular routes.

### Individuals With Immune Response Like Subtype C-c Might Be at Higher Risk of TB Reactivation

From the pattern of pathway perturbation, it could be seen that samples in C-c showed perturbation in the highest number of pathways in the identified IPLTB. We sought to test if it corresponded to the state of the disease or possibility of progression into active TB. 46 LTBI samples with information on confirmed progression into active disease were available in two datasets, GSE107994 and GSE79362. The perturbation pattern of IPLTB in these samples was analyzed using the same network analysis method and their clustering pattern was observed. Although the samples did not exclusively belong to any of the subtypes, a bias towards C-c was indeed observed (**Figure 5C** and **Supplementary Figure 3**). Interestingly, among the non-progressor LTBI samples from GSE79362, a clustering bias was observed for C-d, which had the highest number of non-progressors (60 of the 153) samples (**Figure 5B**). Although, 12 of the 16 genes from the Zak16 signature (18) were present in some of our sample LTBINets, only a few were DEGs in early time points and no specific pattern in cluster membership of those genes was observed, indicating that the Zak16 signature is not repurposable for subtyping LTBI samples. We also studied the membership of genes from other progression signatures, RISK4 (42) and PREDICT29 (43) in IPLTB, but did not find any correlation between the signatures and our pathway-based subtypes.

12 transcriptomic datasets (**Supplementary Table S1.B**) on whole blood from active TB and uninfected cases were also analyzed. Since, gene expression perturbations in active TB condition are significantly homogeneous (**Figure 1A**), the datasets were analyzed as sample pools instead of as individual samples. The perturbation pattern of IPLTB in active TB condition showed that 7 of the datasets showed similarity with the LTBI subtypes, of which 5 resembled C-c (**Figure 1C** and **Supplementary Figure 3**). The rest of the datasets were not similar to any of the subtypes, which can be expected since the immune response in LTBI and active TB conditions vary greatly. This also suggests that the samples in C-c might have higher resemblance in their immune status with active TB state than the other subtypes. From these two observations it can be suggested that the samples in C-c have a higher possibility of the presence of subclinical disease or propensity towards progression into active TB than the samples from any other subtype.

## Discussion

In asymptomatic LTBI cases, the balance between bacterial containment and persistence is caused due to certain immune responses in the host, which prevents the pathogen from multiplying and causing the disease. Although whole blood transcriptomic studies have previously been widely successful in identifying differential gene expression signatures for active TB (7, 13–17), they have not been successful in differentiating between the HC and asymptomatic LTBI populations (7, 13). All of these studies, along with other gene expression meta-analysis have clearly shown that there is no statistically significant differential gene expression between these two (20). Our independent analysis of the datasets here corroborates these findings.

We hypothesized that the specific molecular alterations that enable the host to keep *Mtb* in a latent state are not universally the same in all individuals, and thus the gene expression profiles are also vastly different leading to the observation of no common DEGs across different cohorts. It is possible however, that there are broad similarities at the level of pathways among these individuals, which we have investigated in this work. We adapted the network analysis method from our previous work and have carried out a meta-analysis of the LTBI samples at the level of networks. This allowed us to identify the common key subnetworks and the pathways that are frequently perturbed in LTBI individuals through different gene expression patterns.

**Figure 6:**
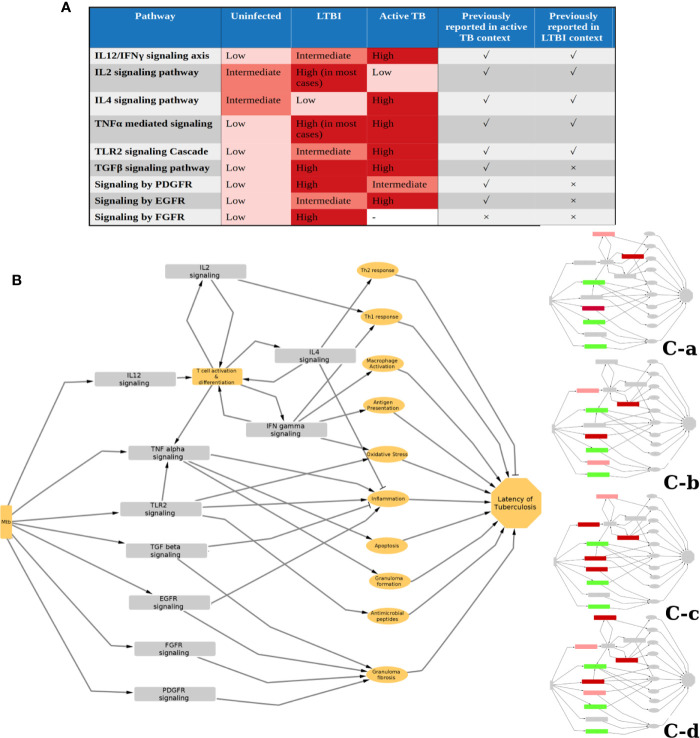
Status and function of IPLTB in uninfected (HC), LTBI and active TB patients. **(A)** The extent of activity of the IPLTB pathways is active TB condition compared to HC and LTBI was analyzed using a similar network analysis method. From this analysis, as well as literature reports, the status of these pathways in the 3 conditions, HC, LTBI and active TB, are summarized in a semiquantitative manner. IL12/IFNγ, TLR2 and EGFR mediated signaling pathways were found to be more active in LTBI compared to HC and further activated in active TB. IL2 and IL4 showed an opposite trend of activity in LTBI and active TB. TGF and TNF were more active in LTBI and active TB compared to uninfected, but the difference between LTBI and active TB cannot be commented upon from our analysis. PDGFR mediated pathway was observed to be more active in both LTBI and active compared to HC, but the extent of activity was higher in LTBI. FGFR mediated signaling was not observed to be one of the top perturbed pathways in active TB condition. The pathways previously reported to have an important function in the context of active and LTBI are marked with a tick, whereas no previous report is marked with a cross. **(B)** The possible effects of the IPLTB on the host immune system are drawn as a simplified schematic. The pathways can be linked to inflammation, macrophage activation, antimycobacterial effects, granuloma formation and fibrosis, etc., which can finally help in maintaining TB latency. The different clusters use some of the pathways from IPLTB, as shown in insets, to achieve TB latency. Red and green correspond to perturbed activities, in comparison to HC. Green denotes the pathways perturbed in all the clusters, whereas red shows pathways perturbed in different clusters. Dark red signifies that most of the members of the cluster show the perturbation whereas light red signifies about half of the members to show the perturbation.

We identified 10 pathways to be commonly perturbed in LTBI samples, which include multiple cytokine mediated signaling pathways, such as IL2, IL4, IFNγ, TNFα, which are known to be involved in the host immune response to active TB. Many of these are also reported to be involved in *Mtb* latency in animal models, as summarized in **Supplementary Table S1.A**. Our networks indicate an increased activity in the IL12 and IFNγ pathways in LTBI. This is in high agreement with previous reports from literature indicating that an increase in the levels of IL12 and IFNγ impart resistance against *Mtb* (44–46). Our method also identified an increase in IL2 in many LTBI cases and a reduction in activity in IL4 signaling, suggesting a high Th1 and a low Th2 cell activity in LTBI condition, as compared to healthy samples. The significance of Th1/Th2 balance and the cytokines IL2 and IL4 in maintaining LTBI has been reported earlier (47–50). Our networks also suggested that the pro-inflammatory cytokine TNFα signaling to be perturbed in most of the LTBI samples as compared to HC. TNFα has been described as a double-edged sword with respect to its role in tuberculosis. While it is known to have a beneficial effect on the host by controlling *Mtb* infection, it can also cause severe tissue damage in active tuberculosis (51). However, in Th1 dominant LTBI, it is known to activate macrophages playing a host protective function (52). This also explains increased LTBI reactivation in patients treated with anti-TNF drugs like infliximab (53, 54). IFNγ and TNFα can also synergistically induce oxidative stress response by macrophages, leading to an antimycobacterial effect (55). Our analysis also shows TLR2 mediated responses to be high in some LTBI individuals (clusters 7-9). TLR2 activation can lead to an induction of an antimicrobial peptide cathelicidin and also induce Th1 cytokine release, which in turn helps in containing mycobacterial infection (56–58). Polymorphisms in TLR2 pathway genes have also been linked to tuberculosis susceptibility (59, 60). Since mycobacterial cell wall components can directly induce TLR2 mediated response (57), it is possible that the extent of TLR2 responses is dependent on the bacterial burden in the individual. These pathways are also perturbed in active TB condition to a different extent as reported in literature as well as observed in our analysis when active TB samples were compared to HC and LTBI conditions following a similar method (**Figure 6A**).

The successful containment of *Mtb* in the host requires a protective granuloma structure with a fibrotic capsule and a necrotic center that can restrict the pathogen (61–63). In addition to TLR2 and cytokine signaling, our analysis indicated TGFβ, EGFR, PDGFR and FGFR mediated signaling responses to be highly active in LTBI. TGFβ levels are also known to be high in active TB and are required for intracellular survival of *Mtb* (**Figure 6A**) (64, 65). Drug repurposing studies have identified that Gefitinib, an EGFR inhibitor, restricts *Mtb* growth, providing support for the involvement of the EGFR signaling pathway in tuberculosis infection (66). High activities of these pathways are known to be beneficial to the pathogen and thus are associated with active TB (67). Our analysis suggests that an increase in the activity of these pathways as compared to HC is associated with LTBI. Further support for the involvement of the EGFR pathway comes from an observation that Erlotinib, an EGFR inhibitor leads to reactivation of LTBI (41). All of these pathways can cause collagen deposition and fibrosis (68–72). Put together, these indicate that an increase in activity of the four pathways compared to HC might help in increasing fibrosis of granuloma and restricting *Mtb* dissemination in LTBI cases. EGFR signaling can increase proliferation of anti-inflammatory M2 type macrophages in tumor-like environments and adoptive transfer of M2 macrophages have been reported to attenuate *Mtb* infection (73, 74). It is possible that a moderately enhanced activity through this pathway could result in LTBI, while a highly enhanced activity is associated with active TB (**Figure 6A**).

Although all of these pathways can be involved in controlling *Mtb* infection, we observed that only a subset of these pathways are perturbed in any LTBI individual. We could divide the total LTBI population into 4 large subtypes with a few outliers based on different molecular routes taken by the individual immune system to achieve latency. It shows that although there is a similarity between the host responses in LTBI individuals, there also exists heterogeneity in exactly how the host system controls the infection. All of the identified pathways can lead to inflammation regulation and antimicrobial effect or granuloma formation (**Figure 6B**). Therefore, the host immune system can achieve latency of *Mtb* by modulating only a combination of these, giving rise to different possible molecular routes to latency (**Figure 6B**). We also observed that LTBI progressors and active TB samples have a higher tendency of being clustered with C-c than the others. Although clinical information on the progression time-course was not available for the other LTBI samples in discovery and validation datasets, this clustering bias of known progressors and active TB cases might indicate that the other LTBI samples belonging to cluster C-c have a higher propensity for subclinical TB or reactivation. Among the four subtypes, C-c shows perturbation in the maximum number of pathways from IPLTB, being the only subtype to show perturbations in both TLR2 mediated signaling and IL4 mediated signaling. Also, C-c is the only subtype that shows perturbed IL4 signaling in all the samples. Increased TLR2 mediated signaling could be caused by a comparatively higher bacterial burden in this subtype as TLR2 can recognize Mtb antigens and it might be related to a higher chance of reactivation of LTBI. Repression of IL4 signaling might play a crucial role in such cases as IL4 can promote pathogenesis, and is therefore repressed in all the progressor LTBI samples before reactivation. Overall, the immune status of the samples in C-c varies the most from uninfected conditions. It is suggestive of LTBI cases with a higher chance of progression into active disease having more perturbations in their immune system even at time points much before TB incidence. This information can provide insights into immune response responsible for susceptibility towards LTBI reactivation and also aid in clinical decision making for personalized therapy of LTBI.

To the best of our knowledge, this is the first systematic analysis that compares the LTBI transcriptomes with those of uninfected individuals that provides an insight into the immune response of the LTBI condition. These identified pathways could possibly be further explored as potential preventive targets for LTBI reactivation in future. This knowledge can also be useful in taking a cautious approach while treating any LTBI subject with drugs targeting these pathways for other diseases.

### Equations

For active precision network,

(1) $$NW_{i}=FC(i)=\frac{\left(Expression\;of\;gene\;’i’\;in\;one\;LTBI\;sample\right)}{\left(Median\;expression\;of\;gene\;’i’\;in\;uninfected\;samples\right)}$$

For repressed precision network,

(2) $$NW_{i}=FC(i)=\frac{\left(Median\;expression\;of\;gene\;’i’\;in\;uninfected\;samples\right)}{\left(Expression\;of\;gene\;’i’\;in\;one\;LTBI\;samples\right)}$$

Where, NW is node weight and FC is fold change.

The edge weight (EW) between nodes ‘i1’ and ‘i2’ is calculated based on the node weights as,

(3) $$EW_{i_{1}i_{2}}=\frac{1}{\sqrt{NW_{i_{1}}\times NW_{i_{2}}}}$$

(4) $$Path\;Cost=\frac{\sum_{i=1}^{n}EW_{i_{1}i_{2}}}{Number\;of\;nodes\;in\;path}$$

## Nomenclature

EGFR, Epidermal growth factor receptor; FGFR, Fibroblast growth factor receptor; IFNγ, Interferon gamma; IL, Interleukin; HC, Healthy (Uninfected) control; LTBI, Latent tuberculosis infection; *Mtb*, *Mycobacterium tuberculosi*s; PDGFR, Platelet derived growth factor receptor; TB, Tuberculosis; TGFβ, Transforming growth factor beta; TLR, Toll like receptor; TNFα, Tumor necrosis factor alpha

## Data Availability Statement

Publicly available datasets were analyzed in this study. This data can be found here: https://www.ncbi.nlm.nih.gov/geo/ under the IDs GSE19439, GSE19444, GSE28623, GSE107993, GSE107994, GSE84076,GSE40553, GSE79362, GSE37250, and GSE101705. Codes used for analysis in this work are available at https://github.com/chandralab-iisc/LTB_immune_Subtype.

## Author Contributions

NC conceptualized, designed, and supervised the study. UB performed data curation, methodology, analysis, and interpretation. PB performed data curation and methodology. AS provided supervision and critical insights. UB wrote the first draft of manuscript. All authors revised and approved the submitted manuscript.

## Funding

The authors thank the Department of Biotechnology, Government of India for providing the funding for this study in the form of a DBT-IISc partnership grant.

## Conflict of Interest

NC is a co-founder of the companies qBiome Research Pvt Ltd and HealthSeq Precision Medicine Pvt Ltd. They had no role in this manuscript.

The remaining authors declare that the research was conducted in the absence of any commercial or financial relationships that could be construed as a potential conflict of interest.
