# Supplementary material for: Immune Subtyping in Latent Tuberculosis
Source: Front Immunol. 2021 Apr 7;12:595746. doi: 10.3389/fimmu.2021.595746 (PMC8059438; doi:10.3389/fimmu.2021.595746)
Supplement: Supplementary file 1 [file DataSheet_1.pdf]

Supplementary Table S1.A: Selected previous literature reports suggesting role of the 10 identified IPLTB in latent or active tuberculosis.

Pathway	Literature report in context of LTB or TB	Reference
IL12/ IFNγ axis	IL12 produced by macrophages after *Mtb* endocytosis induces T-cells activation into CD4+ and CD8+ cells, which can secrete IFNγ. Both IL12 and IFNγ have been implicated in promoting host resistance to *Mtb*.<br>Mutations in several genes in the IL12/IFNγ axis have been implicated in the rare congenital disease known as Mendelian susceptibility to mycobacterial diseases (MSMDs).	[Bustamante et al, 2007, deJong et al, 1998, Serbina and Flynn, 2001]
IL2 mediated signaling	IL2 increases cellular immunity against TB and promotes granuloma formation and is reported to have a high level in mice with latent TB. LTB patients have been shown to have high levels of TH1 cytokines, including IL2.	[Demissie et al, 2004, Howard and Zwilling, 1999]
IL4 mediated signaling	Increased production of the anti-inflammatory cytokine IL4 can promote pathogenesis of pulmonary TB.<br>Increased amounts of IL4 have been implicated in LTBI reactivation in health care workers.<br>Increased expression of IL4 antagonists have been shown to be high in LTBI cases.	[Demissie et al, 2004, Howard and Zwilling, 1999, Ordway et al, 2004]
TLR2 mediated signaling	TLR2 is known to recognize patterns in *Mtb* cell surface and induce Th1 cell response by cytokine secretion. MyD88 deficiency can cause hypersusceptibility to *Mtb* infection in mice. Multiple reports have shown polymorphisms in TLR2 gene to be associated with susceptibility to TB.	[Byun et al, 2012, Sanchez et al, 2010, Thoma-Uszynski et al, 2001]
TNFα mediated response	TNFα can help in granuloma formation and maintenance of *Mtb* dormancy in humans, at the same time it is known to cause tissue damage and promotion of *Mtb* growth in monocytes.	[Hernandez-Pano and Rook, 1994, Mootoo et al,

	Anti-TNF therapy has also been reported to reactivate LTBI in clinical studies.	2009, Shim 2014, Wallis et al, 2004]
PDGFR signaling pathway	PDGF can be linked to delayed type hypersensitivity response and fibrotic reaction in pulmonary TB.	[Klinkhammer et al, 2018, Wangoo et al, 1993]
EGFR signaling pathway	EGF, the ligand for the ERBBs, has receptors on *Mtb* surface as well and can help in bacterial growth within macrophages.<br>A case study showed the EGFR inhibitor erlotinib prescription to a lung cancer patient to reactivate LTBI.	[Bermudez et al, 1996, Lee et al, 2017]
FGFR signaling pathway	No direct report on action of FGFR in TB	
TGFβ mediated signaling	TGFβ is excessively produced in active TB.<br>It is suggested as a potential target to increase bacterial clearance by promoting cytotoxic T-cell activity.	[DiFazio et al, 2016, Jayaswal et al, 2010, Wu et al, 2012]

Supplementary Table S1.B: Deatils of datasets with active TB and uninfected samples used in analysis of Section 4.4

GEO ID	Platform	Uninfected Samples	Active TB Samples	Geographic Location	Age Group	Reference
GSE19435	Illumina GPL6947	12	7	UK	Adult	Berry et al, 2010
GSE19439	Illumina GPL6947	12	13	UK	Adult	Berry et al, 2010
GSE19444	Illumina GPL6947	12	21	UK	Adult	Berry et al, 2010
GSE28623	Agilent GPL4133	37	46	The Gambia	Adult	Maertzdorf et al, 2011
GSE34608	Agilent GPL6480	18	8	Germany	Adult	Maertzdorf et al, 2012
GSE42825	Illumina GPL10558	23	8	UK	Adult	Bloom et al, 2013
GSE42826	Illumina GPL10558	52	11	UK	Adult	Bloom et al, 2013
GSE42830	Illumina GPL10558	38	16	UK	Adult	Bloom et al, 2013
GSE56153	Illumina GPL6883	18	18	Indonesia	Adult	Ottenhoff et al, 2012
GSE83456	Illumina GPL10558	61	45	UK	Adult	Blankley et al, 2016
GSE84076	Illumina Hiseq GPL16791	12	8	Brazil	Adult	De Araujo et al, 2016
GSE107731	Affymetrix GPL15207	3	3	China	Adult	-

References:

1. Berry, M. P. R., Graham, C. M., McNab, F. W., Xu, Z., Bloch, S. A. A., Oni, T., et al. (2010). An interferon-inducible neutrophil-driven blood transcriptional signature in human tuberculosis. Nature 466, 973–977. doi:10.1038/nature09247
2. Maertzdorf, J., Ota, M., Repsilber, D., Mollenkopf, H. J., Weiner, J., Hill, P. C., et al. (2011). Functional correlations of pathogenesis-driven gene expression signatures in tuberculosis. PloS One 6, e26938. doi:10.1371/journal.pone.0026938
3. Maertzdorf, J., Weiner, J., Mollenkopf, H.-J., Network, T., Bauer, T., Prasse, A., et al. (2012). Common patterns and disease-related signatures in tuberculosis and sarcoidosis. *Proceedings of the National Academy of Sciences* 109, 7853–7858. doi:10.1073/pnas.1121072109.
4. Bloom, C. I., Graham, C. M., Berry, M. P. R., Rozakeas, F., Redford, P. S., Wang, Y., et al. (2013). Correction: Transcriptional Blood Signatures Distinguish Pulmonary Tuberculosis, Pulmonary Sarcoidosis, Pneumonias and Lung Cancers. *PLoS ONE* 8. doi:10.1371/annotation/7d9ec449-aee0-48fe-8111-0c110850c0c1.

5. Ottenhoff, T. H. M., Dass, R. H., Yang, N., Zhang, M. M., Wong, H. E. E., Sahiratmadja, E., et al. (2012). Genome-Wide Expression Profiling Identifies Type 1 Interferon Response Pathways in Active Tuberculosis. *PLoS ONE* 7. doi:10.1371/journal.pone.0045839.
6. Blankley, S., Graham, C. M., Turner, J., Berry, M. P. R., Bloom, C. I., Xu, Z., et al. (2016). The Transcriptional Signature of Active Tuberculosis Reflects Symptom Status in Extra-Pulmonary and Pulmonary Tuberculosis. *Plos One* 11. doi:10.1371/journal.pone.0162220.
7. Araujo, L. S. D., Vaas, L. A. I., Ribeiro-Alves, M., Geffers, R., Mello, F. C. Q., Almeida, A. S. D., et al. (2016). Transcriptomic Biomarkers for Tuberculosis: Evaluation of DOCK9. EPHA4, and NPC2 mRNA Expression in Peripheral Blood. *Frontiers in Microbiology* 7. doi:10.3389/fmicb.2016.01586.
