# Supplementary figures and images for: Immune Subtyping in Latent Tuberculosis
Source: Front Immunol. 2021 Apr 7;12:595746. doi: 10.3389/fimmu.2021.595746 (PMC8059438; doi:10.3389/fimmu.2021.595746)

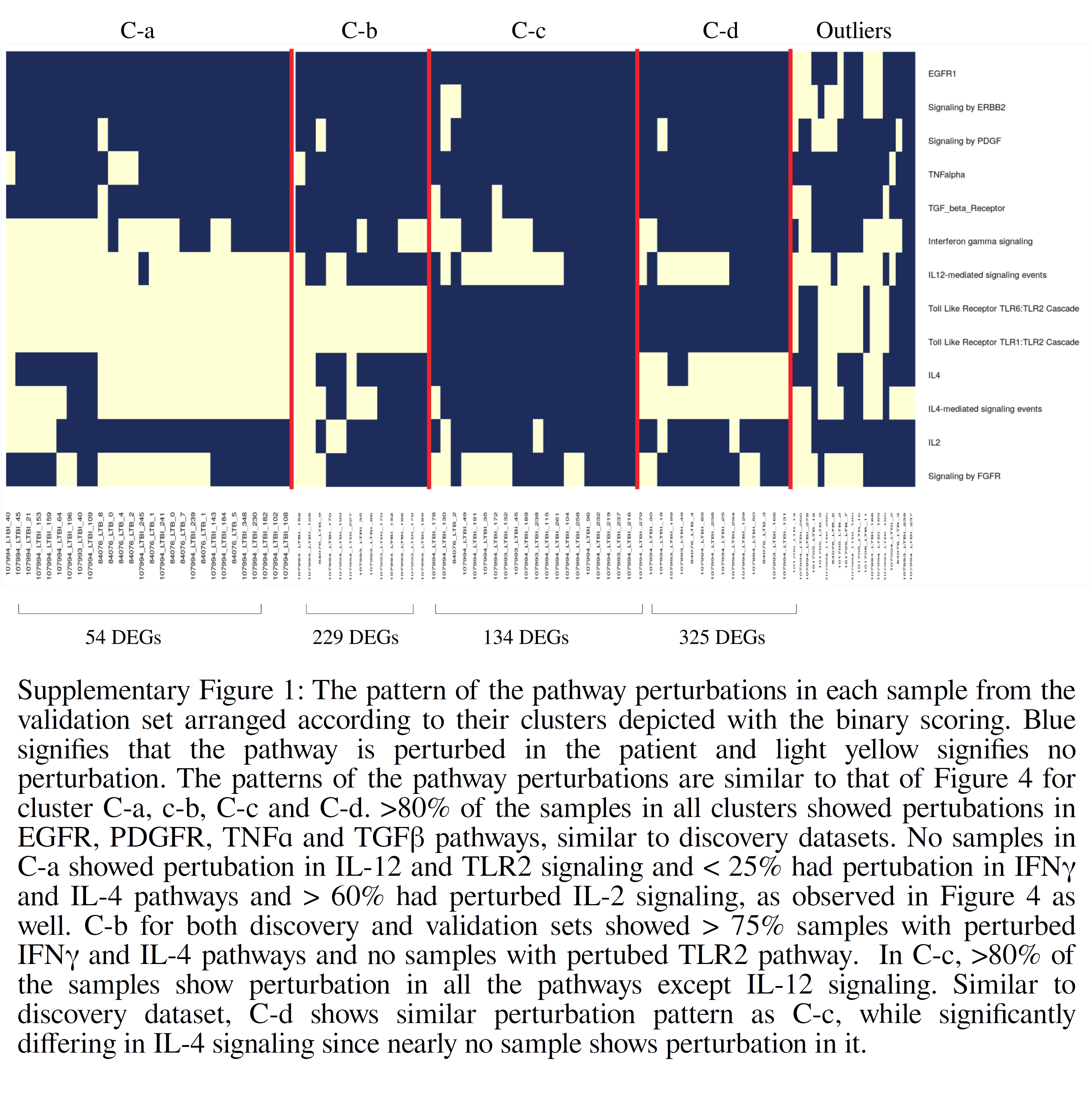

Supplement: Supplementary file 3 [file Image_1.tiff]

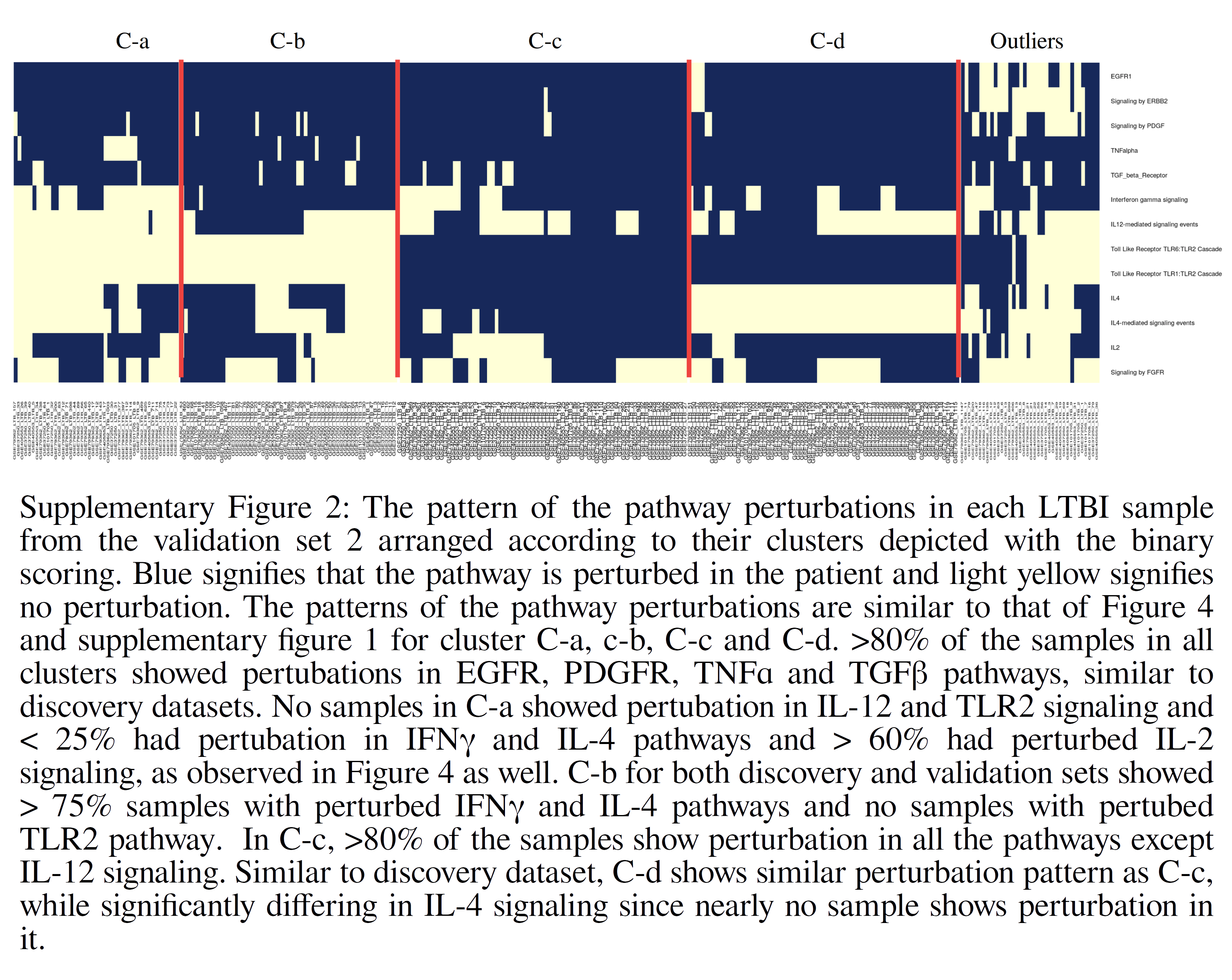

Supplement: Supplementary file 4 [file Image_2.tiff]

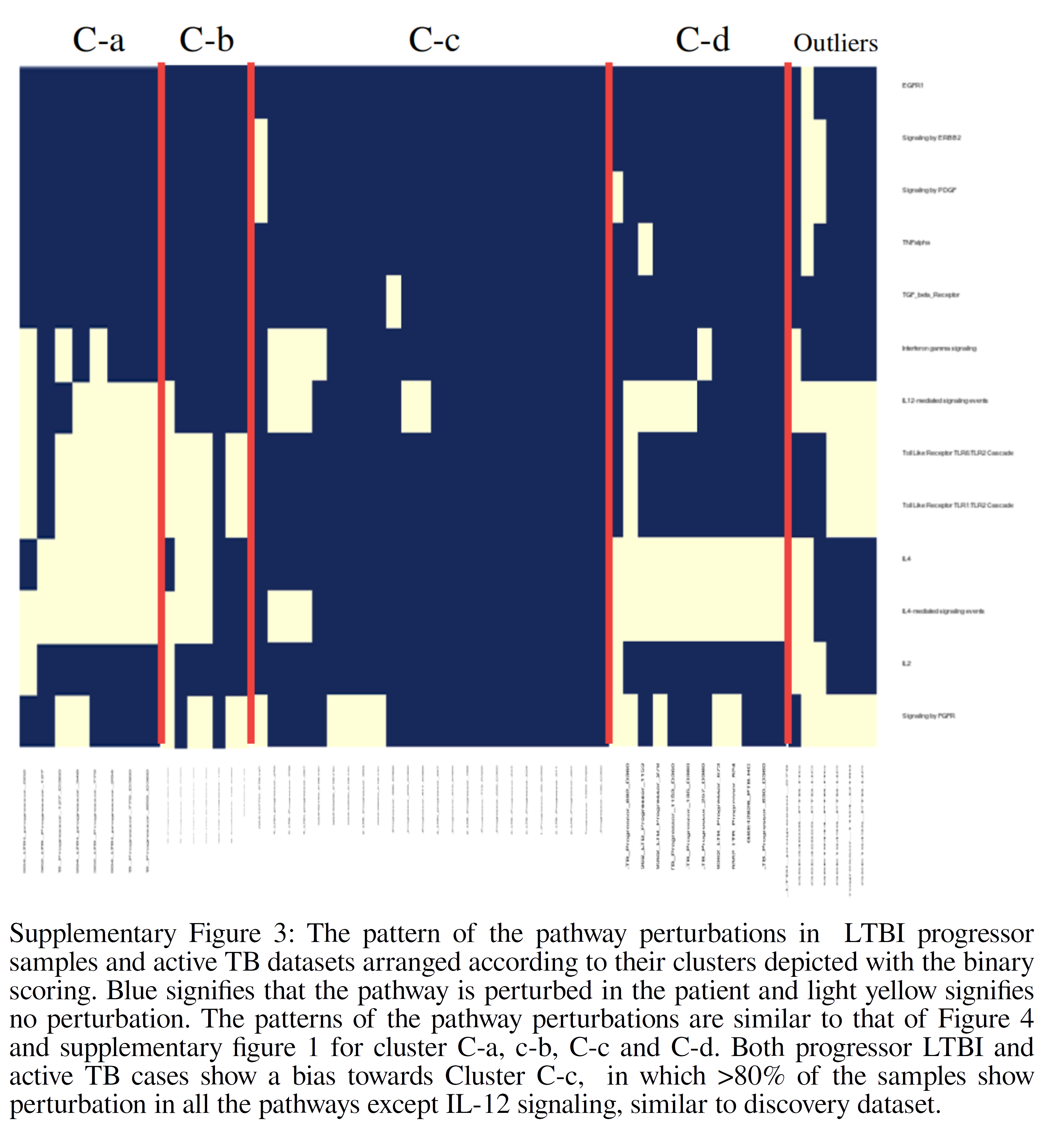

Supplement: Supplementary file 5 [file Image_3.tiff]
